# Intra-abdominal testicular teratoma complicated by torsion: A case report

**DOI:** 10.1016/j.eucr.2025.103299

**Published:** 2025-12-01

**Authors:** Majd Oweidat, Saad Halabi, Raed A.H. Alhashash, Ahmad Hijazi, Haya Taha, Fawaz Halabi, Afnan A. Radaydeh

**Affiliations:** a College of Medicine, Hebron University, Hebron, West Bank, Palestine; b Palestine Red Crescent Specialized Hospital – Hebron, PRCS, Hebron, West Bank, Palestine; c Women's Union Hospital, Nablus, West Bank, Palestine; d Department of Surgery, Rafidia Surgical Hospital, Nablus, West Bank, Palestine; e Department of Radiology, Princess Alia Hebron Governmental Hospital, Hebron, West Bank, Palestine; f Department of Surgery, Princess Alia Hebron Governmental Hospital, Hebron, West Bank, Palestine

**Keywords:** Cryptorchidism, Testicular torsion, Prepubertal teratoma, Case report

## Abstract

A 1-year-old boy with a known left undescended testis presented with 7 days of fever, irritability, and progressive lower-abdominal distension. He was febrile and tachycardic with a firm lower-abdominal mass and an empty left hemiscrotum; white blood cells 17 × 10^9^/L, C-reactive protein 200 mg/L, platelets 680 × 10^9^/L, hemoglobin 6.8 g/dL. Ultrasound showed a heterogeneous avascular intra-abdominal mass; computed tomography showed a large cystic lesion containing fat and calcifications with a left pedicle “whirlpool sign”. Urgent laparotomy with detorsion and excision revealed prepubertal-type testicular teratoma. This case highlights torsion's abdominal presentation in cryptorchidism and the value of prompt surgery and timely orchiopexy.

## Highlights

• Rare torsion of intra-abdominal prepubertal testicular teratoma in an infant.
• Missed undescended testis presenting with abdominal distension and fever.
• Imaging revealed fat, calcifications, and twisted pedicle “whirlpool sign”.
• Urgent laparotomy confirmed torsion and complete excision was curative.
• Emphasizes early orchiopexy to prevent torsion and related complications.

## 1 Introduction

Testicular torsion is a time-critical urologic emergency in which twisting of the spermatic cord compromises testicular perfusion, demanding rapid recognition and operative management to avoid loss of the gonad.^1^ Although classically a disorder of adolescents, torsion can occur at any age and may present with lower abdominal rather than scrotal symptoms.^1^ Ultrasound (US) is the first-line modality for scrotal pathology.^1^

Cryptorchidism is the most common congenital anomaly of the male genitalia; approximately 3 % of term and up to 30 % of preterm boys are affected at birth, but most testes descend spontaneously during the first three postnatal months, leaving a true prevalence near 1 % beyond infancy.^2^ Persistent undescended testis (UDT) is associated with increased risks of subfertility, malignancy, and testicular torsion, and current guidance recommends orchiopexy between 6 and 18 months of age to prevent these risks.^3^^,^^4^ Torsion of an intra-abdominal testis is uncommon but clinically important because it may present with nonspecific systemic or abdominal signs in infants with known or missed UDT.^2^

Among germ-cell neoplasms, testicular teratoma spans a biologic spectrum. The prepubertal-type teratoma tends to be benign, and non-metastatic, whereas postpubertal teratoma is typically GCNIS-related and has metastatic potential.^5^

In this article, we describe a rare entity of 1-year-old boy with a missed left UDT who found to have a left intra-abdominal prepubertal-type testicular teratoma complicated by pedicle torsion.

## 2 Case presentation

A 1-year-old male infant was brought by his parents to the emergency department with a 7-day history of intermittent fever measured at home up to 39 °C (rectally), irritability with inconsolable crying, and progressive lower abdominal distension noted by the family. He had previously been evaluated at a local outpatient clinic and treated for an upper respiratory tract infection without improvement.

The child was known to have a left UDT for which an elective orchiopexy had been scheduled approximately two months earlier but was missed. No abdominal mass had been documented during previous routine examinations, and the family reported that the distension appeared only in the week prior to presentation, suggesting recent rapid enlargement. Pregnancy and birth were uncomplicated after a full-term spontaneous vaginal delivery, growth and developmental milestones were appropriate for age, he was mixed-fed and tolerating table foods, immunizations were up to date, no chronic medications or known drug allergies, and there was no history of preceding abdominal trauma, vomiting, diarrhea, constipation, hematuria, rash, prior hospitalizations, surgeries, or relevant family or social history. Apart from presenting complaints, review of systems was negative for respiratory distress, poor feeding, lethargy, seizure-like activity, bleeding, or bruising.

On arrival he appeared ill, irritable, and uncomfortable with persistent crying; he was febrile, pale, and mildly dehydrated, with no dysmorphic features. Vital signs recorded a heart rate of 200 beats/min, respiratory rate of 40 breaths/min, temperature of 38.5 °C, and normotension for age. Abdominal examination revealed a distended lower abdomen with a firm palpable mass and intermittent guarding; the left hemiscrotum was empty and the right testis was normally descended. Aside from these findings, examination of the other systems was unremarkable.

**Fig. 1:**
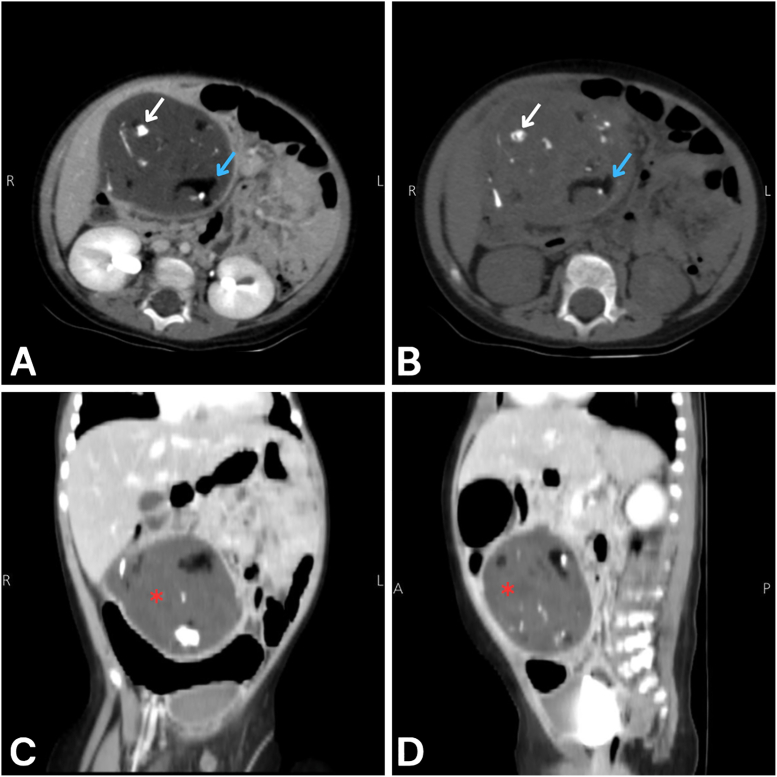
A: Axial abdominal CT without contrast. B: Axial abdominal CT with IV contrast. C: Coronal abdominal CT without IV contrast. D: Sagittal abdominal CT without IV contrast. The images show a large, well-defined, non-enhancing heterogeneous cystic mass (red asterisk) with areas of fat density (blue arrow) and coarse calcifications (white arrow), consistent with a testicular teratoma showing ischemic changes.

**Fig. 2:**
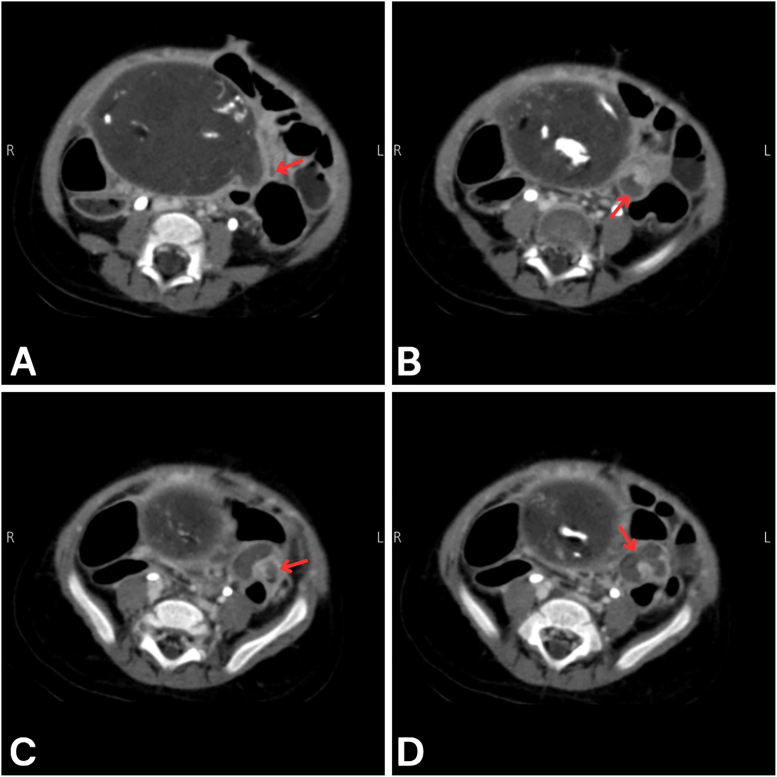
Sequential axial abdominal CT images with IV contrast showing the "whirlpool sign". A: The superior aspect of the twisted vascular pedicle (red arrow) appearing adjacent to the large cystic teratoma. B: Progression of the spermatic cord rotation (red arrow), beginning to form a spiral pattern. C: Further progression of the spermatic cord rotation (red arrow). D: The inferior extent of the twisted vascular pedicle (red arrow).

An abdominal US performed, which showed a well-defined heterogeneous intra-abdominal mass containing calcifications and showing no internal vascular flow on color Doppler ultrasonography, prompting referral. Scrotal US showed an empty left hemiscrotum with a normally vascularized right testis. Because US could not determine its exact origin or vascular supply, an urgent abdominal computed tomography (CT) without and with intravenous (IV) contrast was obtained to further characterize the lesion and assess for features suggestive of teratoma or torsion. As shown in Fig. 1, Fig. 2, CT scan revealed a large, well-defined heterogeneous cystic mass measuring approximately 8.8 × 6.7 × 7.7 cm. The lesion contained macroscopic fat and coarse calcifications with no appreciable enhancement. It was in the right lower abdomen, distinct from adjacent bowel loops, and was supplied by a left-sided vascular pedicle that crossed the midline and showed the characteristic ‘whirlpool sign.’ Overall, the radiologic findings were consistent with a left intra-abdominal testicular teratoma complicated by torsion and ischemic changes.

Initial laboratory studies on admission showed white blood cell count (WBCs) 17 × 10^9^/L, C-reactive protein (CRP) 200 mg/L, platelet count 680 × 10^9^/L, and hemoglobin 6.8 g/dL; other serum biochemistry values were within the laboratory reference intervals. Given the concern for torsion of an intra-abdominal testis, the patient was taken for urgent operative exploration without delay. Packed red blood cells (PRBCs) were initiated preoperatively for severe anemia and continued intraoperatively as needed; isotonic IV fluids were administered to correct dehydration.

**Fig. 3:**
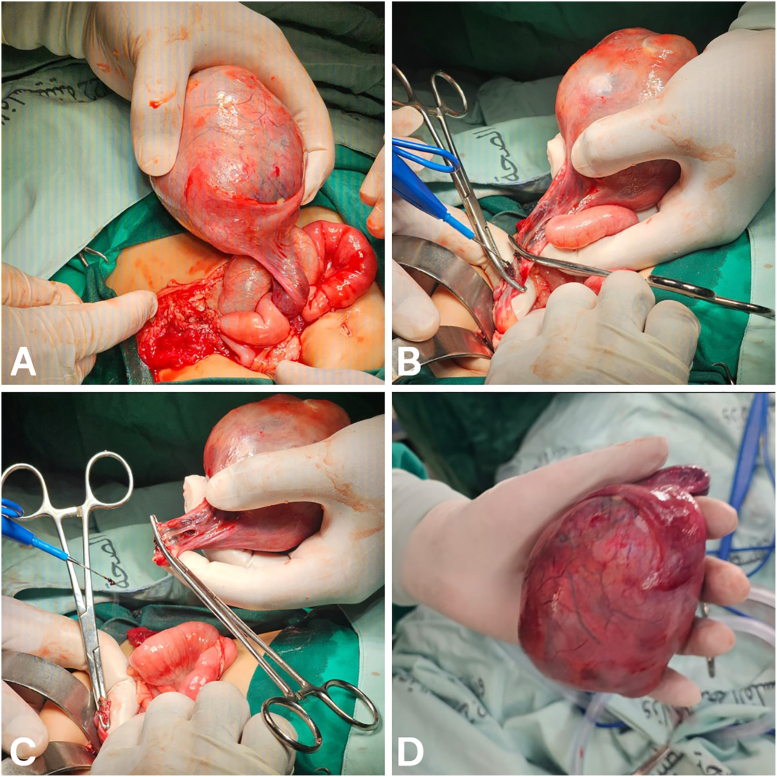
Intraoperative findings during exploratory laparotomy. A: Initial delivery and mobilization of the inflamed, large intra-abdominal testicular mass. B: Isolation of the vascular pedicle. C: Ligation and clamping of the spermatic cord pedicle following detorsion. D: Gross view of the completely excised specimen held in hand, revealing a large, well-circumscribed, and firm mass.

The patient underwent urgent exploratory laparotomy, which revealed an inflamed intra-abdominal testicular mass arising from the left spermatic cord and torsed approximately three complete turns around its pedicle (Fig. 3); detorsion was performed, the mass was ligated at the level of the spermatic cord and excised, and the appendix, which was diffusely reactive and inflamed, was removed by appendectomy. Histopathological examination reported a prepubertal testicular teratoma with foci of necrosis; the resection margin showed focal congestion. Step-sectioning of the testis showed a multicystic cut surface occupying nearly all parenchyma, containing firm brown tissue, hair, and hard bony components with blood-filled cystic spaces.

Postoperatively, the child remained hemodynamically stable and afebrile, tolerated oral intake, and showed down-trending inflammatory markers with normalization of the WBCs count and CRP; he was discharged home in good condition with arrangements for routine outpatient follow-up.

## 3 Discussion

This case highlights an infant with known UDT who developed a large intra-abdominal mass radiologically consistent with teratoma, supplied by a left-sided pedicle with a whirlpool appearance, and confirmed at laparotomy as a torsed intra-abdominal testis harboring prepubertal-type teratoma.

Torsion results first obstructing venous outflow and then arterial inflow, progressing to ischemia and necrosis if untreated; testicular viability declines sharply beyond the early hours of symptom onset.^1^ Although abrupt scrotal pain is classic, infants and young children may manifest irritability, fever, and abdominal findings rather than focal scrotal signs, and US cannot safely delay operative decision-making when clinical suspicion is high.^6^ Our patient's systemic inflammatory response with abdominal mass and Doppler findings supportive of avascularity and a twisted pedicle justified urgent exploration.

Most cryptorchid testes descend, but persistent UDT after six months warrants referral and surgical correction by 6–18 months to reduce the complications.^3^^,^^4^ Intra-abdominal testes constitute a subset of nonpalpable UDT and may evade detection on routine US; thus, reliance on imaging to “rule out” an intra-abdominal gonad is discouraged in guidelines, with diagnostic laparoscopy often definitive when nonpalpable.^7^ This child had a known missed orchiopexy, and torsion of an intra-abdominal UDT is a recognized, if infrequent, complication within the cryptorchidism risk spectrum.^8^

Typical prepubertal teratoma presents as a painless scrotal mass; by contrast, our patient had systemic inflammation and abdominal distension due to torsion of an intra-abdominal gonad.^5^ Grossly and on imaging, teratomas are heterogeneous and frequently contain cystic spaces, fat, calcifications, cartilage, or bone.^5^ These features are mirrored in this case's CT and histopathology results. In the prepubertal population, testicular teratomas are typically benign, non-GCNIS–related tumors with excellent outcomes following complete surgical excision. They commonly occur in infants and toddlers and usually present as well-circumscribed heterogeneous masses^9^. On ultrasound, prepubertal teratomas often demonstrate a mixture of cystic and solid components, echogenic foci from calcification, and variable echogenic fat-containing areas. These features help distinguish teratomas from other pediatric testicular lesions. Unlike postpubertal teratomas, which may behave aggressively and metastasize, the prepubertal type rarely spreads and is generally cured with surgery alone^9^.

The co-occurrence of intra-abdominal prepubertal-type teratoma and acute torsion in a 1-year-old with missed UDT encapsulates a highly unusual but clinically significant presentation pathway for a common emergency. Torsion typically presents with scrotal symptoms, but in cryptorchidism the presentation may be intra-abdominal, mimicking other surgical conditions and potentially delaying definitive care. Radiologic recognition is highly suggestive of a torsed gonadal tumor and supports rushed surgical management.

Point-of-care and formal US are first-line for suspected torsion, but when the testis is nonpalpable or intra-abdominal, cross-sectional imaging can show teratomatous elements (fat/calcification) and pedicle torsion; however, management must remain time-sensitive, with surgical exploration prioritized when torsion is suspected.^1^^,^^6^ In torsion, contralateral fixation is standard when bell-clapper anatomy is suspected; in neonates, bilateral exploration is typical, whereas in older infants/children practice varies with intraoperative findings and institutional protocols.^1^ For prepubertal-type teratoma confined to the testis or arising from an intra-abdominal UDT, complete surgical removal is definitive; adjuvant chemotherapy is not indicated for pure teratoma and is generally ineffective.^10^

In clinical practice, it is essential to maintain a high index of suspicion for torsion when a child with known or suspected UDT presents with abdominal pain, distension, or systemic inflammatory signs, as scrotal findings may be minimal. Operative exploration should not be delayed for additional tests when torsion is likely, since imaging serves to support but should not replace timely surgical intervention. Adhering to the recommended timing of orchiopexy between 6 and 18 months is vital to reduce the risks of torsion, malignancy or infertility. In cases of prepubertal-type teratoma, complete surgical excision is generally curative.

## 4 Conclusion

In summary, this case highlights the rare coexistence of intra-abdominal testicular teratoma and torsion in a cryptorchid infant.

## CRediT authorship contribution statement

**Majd Oweidat:** Writing – review & editing, Writing – original draft, Visualization, Validation, Supervision, Software, Resources, Project administration, Methodology, Investigation, Data curation, Conceptualization. **Saad Halabi:** Resources, Methodology, Investigation, Data curation. **Raed A.H. Alhashash:** Supervision, Conceptualization. **Ahmad Hijazi:** Investigation, Data curation. **Haya Taha:** Writing – original draft, Visualization. **Fawaz Halabi:** Data curation. **Afnan A. Radaydeh:** Data curation.

## Informed consent

Written informed consent was obtained from the patient's parents/legal guardian for publication and any accompanying images. A copy of the written consent is available for review by the Editor-in-Chief of this journal on request.

## Ethical approval

Ethical approval was not required for this study in accordance with local ethics committees on reporting single patient cases.

## Funding

The author(s) received no financial support for the research, authorship, and/or publication of this article.

## Declaration of competing interest

The author(s) declared no potential conflicts of interest with respect to the research, authorship, and/or publication of this article.
